# Death and Camphene

**Published:** 1860-03

**Authors:** 


DEATH AND CAMPHENE.—We copy the following from the New York Observer, a religious paper, and invite to it the serious attention of the reader:—

Before the recent terrible calamity at Lawrence, Mass., which received its most horrid feature from the introduction of a camphene lamp, we had seen the following statistics by Mr. Merriam, of Brooklyn. They show the number of deaths from camphene and other similar burning fluids, as mentioned in the papers since July 22, 1850:—

| | Killed. | Injured. |
|---|---|---|
| 1850 | 2 | 10 |
| 1851 | 23 | 49 |
| 1852 | 15 | 31 |
| 1853 | 28 | 50 |
| 1854 | 55 | 70 |
| 1855 | 40 | 46 |
| 1856 | 65 | 93 |
| 1857 | 59 | 75 |
| 1858 | 54 | 93 |
| 1859 | 83 | 106 |

Whole number since July 22, 1850, 424; injured, 623.

Since the first instant, a fire occurred in Division Street, in this city, occasioned by the bursting of an over-heated lamp of burning-fluid, by which six persons, most of them young children, were burned to death.

But the most horrible occurrence that we have ever seen recorded in connection with the use of these dangerous fluids, is that which followed the falling of the Pemberton Mill at Lawrence. All accounts agree in describing the fire which ensued, by which so many who seemed on the point of being rescued, some of them wounded but others unharmed, were literally roasted to death, as horrible beyond description. The following, taken from one of the latest reports, is said to be the true explanation of its occurrence:—

"*How the fire originated.*—It would seem that the fatal burning of the wrecked building was another '*Camphene Accident.*' The history of the disaster by the fire, now accepted at Lawrence, is, that a man zealous to relieve suffering and save life, endeavored to pass some hot coffee through an aperture into a space where several men and women, unharmed by the crash, were yet imprisoned between the wall and the beams. To find his way safely in for his pitcher, he first introduced his lantern. It met with some hard resisting substance and was knocked from his hand. It broke as it fell, and the camphene with which it was charged of course ignited while it was scattered. The loose cotton took fire immediately, and the terrible consequences are now history."

Is it not time for our legislatures to interpose, to protect by law human life and property, if these destructive fluids cannot be banished from domestic use in any other way? No possible care can prevent frequent disasters, because they occur in unforeseen ways, and not merely by what is called carelessness. The very use of these fluids indicates a degree of carelessness of which it would seem no prudent person could be guilty. They doubtless have their place, but not in the domestic circle, where they are liable at any moment to scatter death.

